# Hepatitis D virus infection in Kermanshah, west of Iran: seroprevalence and viremic infections 

**Published:** 2018

**Authors:** Babak Sayad, Yosra Naderi, Seyed Moayed Alavian, Farid Najafi, Alireza Janbakhsh, Feyzollah Mansouri, Siavash Vaziri, Mandana Afsharian, Fatemeh Norooznezhad

**Affiliations:** 1 *Liver Diseases Research Center, Kermanshah University of Medical Sciences, Kermanshah, Iran*; 2 *Baqiyatallah Research Center for Gastroenterology and Liver Disease, Baqyiatallah University of Medical Sciences, Tehran, Iran*; 3 *School of Public Health, Kermanshah University of Medical Sciences, Kermanshah, Iran *

**Keywords:** Hepatitis D, Prevalence, Viremia, Iran

## Abstract

**Aim:**

This study aimed to determine the seroprevalence and viremic infection of hepatitis delta virus (HDV) in Kermanshah.

**Background:**

Hepatitis delta is one of the most complex viral infections of liver that along with hepatitis B virus could lead to fulminant hepatitis, progressive chronic hepatitis, cirrhosis, and hepatocellular carcinoma.

**Methods:**

Referrals with positive HBs Ag were included and tested for HDV Ab using ELISA. Seropositives were subsequently evaluated for viremia by assaying HDV RNA and HBV DNA using real-time PCR. Viremia-related variables were also assessed.

**Results:**

From 1749 patients included, 30 had positive HDV Ab, which makes HDV seroprevalence 1.7%. Twenty-nine out of 30 seropositives were assayed for viremia. Fourteen cases (48.3%) had positive HDV PCR, 18 (62.1%) had positive HBV DNA. Eight patients (27.6%) had simultaneous replication of HBV and HDV, six (20.7%) only had HDV replication, ten (34.5%) only had HBV replication and five (17.2%) had no replication of either viruses.

**Conclusion:**

Kermanshah seems to be a low prevalent area in Middle East. Viremic HDV infection was lower compared to Europe and Africa, probably due to genetic variations of the hosts or the differences in genotypes or sub-types of hepatitis B and D viruses.

## Introduction

There are 248 million people infected with hepatitis B virus worldwide (1) among whom 15-20 million have been detected to simultaneously carry hepatitis delta virus (HDV) (2) which not only exacerbates the disease course but also complicates the treatment process and worsens the prognosis (3). As the prevalence of hepatitis B virus shows diversities around the world and its epidemiology has changed lately (4), epidemiology of HBV/HDV co-infection also differs geographically. The most co-infected areas have been known to include Mediterranean Basin, Middle East, central and northern Asia, central and West Africa, Amazon Basin and Pacific Islands (2). Hepatitis B vaccination, screening of blood and blood products, screening and post-exposure prophylaxis in health care workers and supply of clean needles have greatly reduced the prevalence of hepatitis B, especially in developed countries in recent years (1). Although epidemiologic control of hepatitis B can decrease hepatitis D virus co-infection prevalence, affecting factors such as migration and intravenous drug use have played critical roles in increasing hepatitis D infection and its revival in developed countries. In developing countries however, availability of laboratory tests has facilitated diagnosis of hepatitis D (5, 6). Iran is located in Middle East, an endemic area of hepatitis D with a seroprevalence of 14.74% in inactive HBsAg carries, 27.8% in chronic hepatitis B and 36.57% in patients with cirrhosis and hepatocellular carcinoma (6). Studies on HBV/HDV co-infection in Iran has been mostly preformed on limited samples resulting in a wide-ranged prevalence; as the reports vary between 0% in Sari (7) to 17.3% in Hamadan (8). Also in HIV-infected patients, prevalence ranges from 19.7% in Shiraz (9) to 31.5% in Kermanshah (10). Moreover, HDV-RNA which is the indicator of viremic infection of hepatitis D has not been reported in most of these studies. Accordingly, this study intended to investigate HDV seroprevalence in HBsAg positive patients and also to detect the viremic cases of hepatitis D by assaying HDV-RNA. 

## Methods

**Study population**

A total number of 1749 serologically confirmed HBsAg positive cases were included in the study. These patients were referred to Liver Diseases Research Center of Kermanshah University of Medical Sciences during November 2004 to July 2016. The diagnosis had been made during blood donations or blood tests performed for different purposes such as screenings before employment, marriage or pregnancy, history of hepatitis B or liver diseases in family, abnormal liver function tests, any evidence of liver disease and etc. Referred cases were from western provinces including Kermanshah, Lorestan, Ilam, Kurdistan, and Hamadan. HDV Ab test was ordered for all patients. For HDV Ab positive patients, qualitative HDV RNA RT-PCR was performed in order to identify viremic cases. To study the related variables regarding viremic hepatitis D, further tests were carried out including HBV DNA level, HBeAg, HBeAb, HCV Ab, and liver function test. Furthermore, in order to assess advanced liver disease (cirrhosis), clinical evaluations and if needed, complementary tests, liver sonography, fibroscan, and liver biopsy were performed. Additionally, standard questionnaires containing demographic and high-risk behaviors characteristics were completed for each patient. 

**Laboratory studies**

Hepatitis B surface antigen (HBsAg), hepatitis C virus antibody (HCV Ab), and human immunodeficiency virus antibody (HIV Ab) were tested using enzyme-linked immunosorbent assay (ELISA) (Pishtazteb Diagnostics®, Iran). Hepatitis B envelope antigen (HBeAg), hepatitis B envelope antibody (HBeAb) and HDV Ab were tested using ELISA (RadimSpA, Rome, Italy). Quantitative HBV DNA PCR was performed using COBAS TaqMan HBV test (Roche Diagnostics) with lower limit of detection equal to 35 copies/mL, according to manufacturer's instructions. Qualitative HDV RNA PCR was done by reverse transcription-polymerase chain reaction (RT-PCR) using Primer Design™ genesig Kit for Hepatitis D Virus (Primerdesign, UK) with a sensitivity of 100 copies/mL. 

**Statistical Analysis**

Statistical analysis was done using Stata software version 13.0 where mean and standard deviation were used for quantitative data and frequency and percentage for qualitative data. For inferential statistics, we used univariate and multivariate logistic regression. Because of small sample size, the exact method was applied. 

## Results

Among 1749 patients with hepatitis B referred to our center from November 2004 to July 2016, 30 positive HDV Ab cases were diagnosed and therefore the hepatitis D seroprevalence in the studied sample was 1.7%. 

From these 30 HBV/HDV co-infected individuals, 16 (53.3%) were male and 14 (46.7%) were female. The age range was from 19 to 82 (mean age 43.07±16.5). Twenty-seven patients were urban (90%) and the other three (10%) were rural. 

Table 1 shows the risk factors in seropositive hepatitis D patients. As it is shown, history of surgery and tattooing were the most frequent risk factors among the patients. 

**Table 1:** Distribution of risk factors and virologic factors in hepatitis D patients

Percentage (%)	Number of patients	Risk Factors
50	15	Surgery
43.3	13	Tattoo
23.3	7	Transfusion
6.7	2	Prison
3.3	1	PWID*

* PWID: People who Inject Drugs

In table 2, patients’ registration time is classified by year. Both HBV and HDV cases are included separately.

**Table 2:** HBV and HDV infected patients’ registration time by year

Year of registration	Number of HBV patients registered	Number of HDV patients registered
2004	93	2
2005	64	0
2006	70	2
2007	69	0
2008	92	0
2009	170	4
2010	156	2
2011	195	3
2012	207	2
2013	206	6
2014	144	5
2015	141	3
2016	112	1

Table 3 demonstrates the virologic markers in hepatitis D seropositive patients. More than 90% of patients had negative HBeAg and only one patient (3.3%) showed positive serologic result for hepatitis C who had history of drug injection. However, further complementary tests for HCV RNA PCR came back negative for this patient.

**Table 3:** Distribution of virologic markers among seropositive hepatitis D patients

Virologic Markers	Number of patients	(%)
HBeAg (+) & HBeAb (-)	2/30	6.7
HBe Ag (-) & HBeAb (+)	26/30	86.7
HBeAg (-) & HBeAb (-)	2/30	6.7
HCV Ab (+)	1/30	3.3
HDV PCR (+)	14/29	48.3
HBV PCR (+)	18/29	62.1

Among 29 patients out of 30 with serologically positive hepatitis D, HBV viral load and HDV PCR were done (one patient passed away due to disease severity following cirrhosis and hepatoma before the molecular tests were performed). Test results showed that 18 out of 29 cases (62.1%) had detectable HBV DNA (with minimum of 20 and maximum of 4.5*10^9^ copies/mL). Viral loads of more than 100,000 and 10,000 copies/mL were observed only in two and six patients, respectively. Positive HDV-PCR was found in 14 out of 29 patients, so 48.3% of study sample had viremic hepatitis delta. In 29 out of 30 HDV seropositive cases for whom HBV and HDV viremia were assayed, eight patients (27.6%) had simultaneous replication of hepatitis B and D, six (20.7%) had hepatitis D replication and undetectable HBV DNA, ten (34.5%) had hepatitis B replication and undetectable HDV RNAand five (17.2%) had no replication of either viruses (Table 4). Among 30 patients under study, six (20%) had the clinical and/or paraclinical indicator of cirrhosis (Table 5).

**Table 4:** Distribution of molecular markers among hepatitis D patients based on infection type.

Infection type	Number (%)	HDV PCR	HBV PCR	HBsAg	HDV Ab
Viremic	8 (27.6)	+	+	+	+
	6 (20.7)	+	-	+	+
Non Viremic	10 (34.5)	-	+	+	+
	5 (17.2)	-	-	+	+
Total	29 (100)				

**Table 5.** Features of patients with viremic and non-viremic hepatitis D infection

Study variable	HDV infection	
	Viremic n (%)	Non Viremic n (%)
Age (>40)	10 (71.4)	4 (28.6)
Sex (Male)	11 (73.3)	4 (26.7)
ALT (Abnormal)	14 (73.7)	5 (26.3)
AST (Abnormal)	11 (55)	9 (45)
HBeAg (+)	10 (40)	15 (60)
HBeAb (+)	10 (40)	15 (60)
HBV DNA (+)	8 (44.4)	10 (55.6)
HBV viral load ≥10000 copies/mL	4 (66.7)	2 (33.3)
HCV Ab (+)	1 (100)	0 (0)
Cirrhosis	5 (100)	0 (0)

**Table 6:** Prevalence of hepatitis D in various populations in Iran

First Author	Publication Year	City	Target population	Number of cases	HDV prevalence%
Taghvaei (7)	2008	Sari	Hepatitis Clinic	167	0
Roshan (11)	2003	Babol	Hepatitis Clinic	546	2
Ghadir MR (12)	2012	Qom	Hepatitis Clinic	48	2
Amini et al (13)	1993	Hamadan	Hepatitis Clinic	123	2.4
Ataei (14)	2011	Esfahan	Hepatitis Clinic	346	2.9
Ziaei (15)	2012	Birjand	Hepatitis Clinic	413	3.1
Roshandel (16)	2007	Golestan	Hepatitis Clinic	139	5.8
Torabi (17)	2002	Tabriz	Hepatitis Clinic	130	6.15
Habibi (18)	2007	Mashhad	Hepatitis Clinic	200	9
Hajiani (19)	2009	Ahvaz	Hepatitis Clinic	1725	11.5
Bakhshipour (20)	2012	Zahedan	Hepatitis Clinic	440	17
Alizadeh (8)	2010	Hamadan	Hepatitis Clinic	81	17.3
Attaran MS (21)	2013	Tehran	Blood Donors	854	2
Kasraian (22)	2012	Shiraz	Blood Donors	185	2.2
Tajbakhsh (23)	2011	Shahrekord	Blood Donors	90	2.2
Motamedifar (9)	2012	Shiraz	HIVinfected	178	19.7
Vaziri (10)	2008	Kermanshah	HIV infected	888	31.5

**Table 7:** Prevalence of hepatitis D in different populations worldwide

Geographic area	Country	First Author	Publication Year	Number of cases/studies	HDV prevalence%
Asia	Iraq (Duhok)	Hussein NR (27)	2015	45	6.6
	Saudi Arabia	Ibrahim Al Traif (29)	2004	67	8.6
	Kuwait	Al-Kandari (30)	1988	254	9
	Pakistan (Punjab)	Zaidi (25)	2010	96	83.3
	Pakistan (Gujrat)	Shah Latika (24)	2012	141	8.5
	Pakistan (Karachi)	Abbasi A (26)	2011	347	28.1
	Korea	Kim HS (31)	2011	940	0.32
	Yemen	Guneid AME (32)	1993	100	2
EMRO	EMRO	Amini N (28)	2013	62 Studies	14.74
Europe	Turkey	Değertekin H (33)	2008	62 studies	12 to 20
	Turkey	SukranKose (34)	2011	3094	2.5
	Belgium	Ho E (35)	2013	800	5.5
	Romania	Gheorghe L (36)	2015	2761	23.1
	Bucharest	Popescu GA (37)	2013	1094	20.4
	Italy	Sagnelli E (38)	1992	1556	23.4
Africa	Egypt (Ismailia)	Gomaa N (39)	2013	170	4.7
	Mauritania	Lunel-Fabiani F (40)	2013	300	30
South America	Brazil	Mendes-Correa MC (41)	2011	86	1.2

## Discussion

Iran is located in Middle East, an endemic area with high prevalence of hepatitis delta (2). Therefore, estimating the disease burden is one of the health priorities in our country. In the present study, 30 individuals out of 1749 HBV-infected patients had positive HDV Ab. In another word, hepatitis delta seroprevalence was 1.7%. There are several studies carried out on hepatitis delta prevalence in Iran (Table 6). These studies are focused on three population groups: the first are the referrals to hepatitis clinics, the second are blood donors and the third includes HIV-infected patients. In the first group, which is more consistent with our study population, co-infection with hepatitisdelta has been reported from 0% in Sari to 17% in Zahedan and 17.3% in Hamadan; where the mean prevalence of hepatitis D in these studies is 8.23%. Although, this prevalence varies greatly throughout different geographical areas with no consistent pattern.

In the second group, blood donors, the prevalence varies between 2% in Tehran and 2.2% in Shiraz and Shahrekord. Since blood donors often do not come from a high risk population, the prevalence of hepatitis D is low in this group. Instead, the third group was consisted of HIV/HBV co-infected patients with high risk behaviors. In Kermanshah (10) and Shiraz (9), HDV prevalence in these patients has been reported as high as 31.5% and 19.7%, respectively, which is not implausible. However, it seems that studies on the first two groups of hepatitis B patients give more reasonable estimation of hepatitis D prevalence. 

In a meta-analysis done by Alavian*et al* in 2011, hepatitis D prevalence was reported 6.61%. This prevalence differs in subpopulations of cirrhotic patients, chronic hepatitis B patients and asymptomatic carriers to be 30.47%, 14.4%, and 4.94%, respectively (3). It is plausible that Kermanshah could be considered a low prevalent area in Iran. Regarding the high number of studied cases and also the population that covers an inclusive range of HBV-infected individuals, the results presented in this study are reliable and referable for Kermanshah.

Hepatitis delta is distributed worldwide while Eastern Mediterranean and Middle East countries are among areas with higher prevalence for this infection (Table 7); wherein the prevalence of hepatitis D in Iran's neighboring countries is significant. Pakistan, Iran’s eastern neighboring country, has the highest prevalence ranging from 8.5% in Gujrat to 28.1% in Karachi and 88.3% in Panjab (24-26). Zahedan, as the capital city of Sistan and Baluchestan neighboring Pakistan, is considered to have a high prevalence of hepatitis D (17%) in Iran (20). On the other hand, western neighbors to Iran have less prevalence of the disease. In Turkey and also Iraq (which is neighbor to Kermanshah), the prevalence has been reported 2% and 6%, respectively (27). These values are slightly higher than the prevalence of 1.7% reported in the present study. Although the availability of diagnosing techniques has made it possible for Middle East countries to detect hepatitis delta infection, concluding about the trend of hepatitis delta virus infection in this area takes longer time. In Europe, hepatitis D prevalence has declined after two decades of its identification as in 2000, it was known as a vanishing disease. Nevertheless, its value in Europe has again started to rise by increased rate of immigration from areas of high prevalence and also its epidemic among injection drug abusers (5). In spite of the low prevalence of hepatitis delta in our study sample, the prevalence of 31.5% in HIV-infected patients who mostly have injection drug use histories (28) is definitely a danger sign of a potential increasing prevalence of hepatitis delta in our country.

For 29 out of 30 patients in our study, HDV-PCR was done and 14 (46.7%) cases were diagnosed positive and viremic. As it is displayed in table 8, the study of viremia in researches on hepatitis delta prevalence is limited. In a study by Ataran *et al.* in Iran Blood Transfusion Organization, five individuals (27.7%) of 18 cases with hepatitis delta were viremic. This value differs in other studies, ranging from 30% in Pakistan to 93% in Italy, with an overall mean of 70%. The low prevalence of viremic infection in our study could be resulted from false positives for HDV Ab or very low or undetectable levels of HDV-RNA. On the other hand, this could have happened because of higher virus clearance rate in our study population as it has also been observed in other studies in Iran (21) and Pakistan (25), as two Asian countries. Accordingly, it seems that variations in hosts genetic and genotypes and sub-types of hepatitis B and D viruses may be of influence. 

In our study, 20% of patients had active replication of hepatitis D with no evidence of hepatitis B replication (table 4), as a result of the suppressive effect of hepatitis D virus which has been already affirmed in other studies (21, 37, 43). However, 26.7% of our patients had simultaneous replication of both viruses with rather high hepatitis B viral load (table 4), as in one patient the viral load was detected as high as four billion copies/mL and also no statistically significant relationship was found between viremic hepatitis D and hepatitis B viral load. Therefore it seems that the reduction of hepatitis B replication by hepatitis delta virus is not an unchanging rule. In a study by Lunel-Fabiani *et al.*, the authors mention the similar point that patients showing simultaneous replication of hepatitis B and D with higher levels of HBV DNA, suffered from more severe liver disease (40). 

30.3% of our patients with non-viremic hepatitis delta infection had detectable hepatitis B viral load (table 4). In the study of G.A.Popescu *et al.*, hepatitis delta seropositive patients with HBV replication alone are considered for treatments with nucleot(s)ide analogues (37). Therefore, in the presence of active liver disease in these patients, where interferon is not possible to be used due to its adverse effects, treatment with nucleot(s)ide analogue drugs is recommended.

Five (16.7%) of our patients had no replication of hepatitis B or D. They had negative HBeAg, positive HBeAb and no advanced liver disease. ALT level was high in only one of them (48 IU/L) whom liver damage was not significant in liver biopsy according to Modified Histologic Activity Index (stage:1 and Grade:1). Accordingly, it is safe to say that this group of patients cleared hepatitis D and are in the inactive carrier state of hepatitis B. So, follow up of hepatitis B is recommended. 

Sex distribution was almost even in our hepatitis delta seropositive patients (16 males and 14 females). Although in most studies, especially on high risk populations, hepatitis D seroprevalence has been reported higher in men than in women due to greater prevalence of high risk behaviors in men (19, 20, 25, 42, 44). In studies on patients referred to hepatitis clinics, sex distribution has also been almost even (8, 15, 26, 34). It is also noteworthy to add that in our study, hepatitis D viremic infection was significantly higher in men (table 5). Although the sample size was low and there are not much similar studies supporting this result (25), it could be due to this general fact that women exhibit stronger immune responses in antigenic challenges; as they are also able to clear HBsAg and produce HBsAb more effectively than men (45, 46). Moreover, we found that hepatitis delta viremic infection is significantly more prevalent at ages above 40 which may be caused by the declined immune response, especially compromised cellular immune responses by aging (47). 

**Table 8:** Prevalence of viremia in seropositive hepatitis delta patients

Country	First Author	Publication Year	Number of HDV seropositive patients	Number of Viremic Patients	HDV Viremia Prevalence (%)
Iran	Attaran MS (21)	2013	18	5	27.8
Pakistan	Zaidi (25)	2010	80	24	30
Korea	Kim HS (31)	2011	3	2	66.6
Mauritania	Lunel-Fabiani F (40)	2013	98	61	62.2
Italy	Niro GA (42)	2010	188	175	93
Bucharest	Popescu GA (37)	2013	223	151	67.7
Romania	Gheorghe L (36)	2015	639	454	71
Total			1246	872	70

We also observed significant relations between hepatitis D viremia with serum alanine amino transferase level and cirrhosis (table 5). Several studies have shown that HBV-infected patients with chronic hepatitis and/or cirrhosis have much higher hepatitis delta seroprevalence than inactive carriers of hepatitis B (3, 6, 28, 33, 48, 49). Moreover, viremic infection of hepatitis D is more prevalent in cirrhotic patients (42, 50).

In our study, two patients (6.7%) had positive HBeAg and negative HBeAb who were viremic for hepatitis D. In two other patients (6.7%), HBeAg and HBeAb were negative but viremic hepatitis D was observed. Among 26 individuals (86.6%) with negative HBeAg and positive HBeAb, 10 (33.3%) had viremic hepatitis D. Although there was no correlation found between HBeAg and viremic hepatitis delta infection, negative HBeAb had a relationship with hepatitis delta viremia. Likewise, in a study by Heidrich *et al.*, there was no relationship reported between HBeAg and hepatitis delta viral load. They also stated that the HBeAg status had no influence on the suppressive effect of HDV on HBV replication (51). 

The limitations to the present study were as follows: first, HDV PCR was carried out qualitatively and HDV viral load was not assayed. Second, HDV genotyping was not done and lastly, HBV risk factors were not obtained from mono-infected patients to compare with HBV/HDV co-infected cases. 

In conclusion, hepatitis delta prevalence in Kermanshah, west of Iran, was 1.7% which is lower than other provinces in Iran and also neighboring countries. Hepatitis D viremia was found in 46.7% of patients which was statistically correlated with ages above 40 years, male sex, high levels of ALT, and cirrhosis. The rate of hepatitis D viremia in our study was less than what has been reported in Europe and Africa which could be resulted from genetic variations of the hosts or differences in genotypes or subtypes of the hepatitis B and D viruses.
